# Airway management by ambulance nurses during out-of-hospital cardiac arrest

**DOI:** 10.1016/j.resplu.2025.100999

**Published:** 2025-06-08

**Authors:** Lotte C. Doeleman, Julian F.F. de Jong, Vera G.M. van Eeden, Patrick Schober, Markus W. Hollmann, Hans van Schuppen

**Affiliations:** a Department of Anesthesiology, Amsterdam University Medical Center, the Netherlands; b Quality of Care, Amsterdam Public Health, Amsterdam, the Netherlands; c Department of Intensive Care, Amsterdam University Medical Center, the Netherlands; d Heart Center, Department of Clinical and Experimental Cardiology, Amsterdam UMC location University of Amsterdam, Meibergdreef 9, 1105 AZ Amsterdam, the Netherlands; e Heart Failure and Arrhythmias, Amsterdam Cardiovascular Sciences, Meibergdreef 9, 1105 AZ Amsterdam, the Netherlands

**Keywords:** Advanced Life Support (ALS), Airway management, Cardiac Arrest, Cardiopulmonary Resuscitation (CPR), Emergency Medical Services (EMS), Ventilation

## Abstract

**Background:**

Adequate airway management in out-of-hospital cardiac arrest (OHCA) is crucial for ventilation and oxygenation. Advanced airway management with a supraglottic airway device (SAD) or endotracheal tube (ETT) follows bag-valve-mask (BVM) ventilation, with emergency front-of-neck access as a rescue-option. EMS protocols on airway management in OHCA have changed over the years and during the COVID-pandemic. This study assessed subsequent changes in airway management and the success of different strategies.

**Methods:**

An observational study was conducted with data from ARREST, 2019–2023. All consecutive adult OHCA patients who had resuscitation attempted by EMS were included. Patients were excluded if helicopter emergency medical services were involved. Changes in devices used intra-arrest and their first-pass success were analyzed.

**Results:**

The proportion of cases with an SAD attempt increased, and with an ETT decreased. The definitive airway device used was an SAD in 59% (95% CI: 57–60%), an ETT in 21% (95% CI: 19–22%), and BVM in 14% (95% CI: 13–15%). First pass success for ETT increased from 53% to 68%. Average SAD first pass success was 93%.

**Conclusion:**

During cardiopulmonary resuscitation (CPR) by ambulance nurses, the use of SADs increased, and that of ETTs decreased. Although ETT first pass success improved, it was lower than the guideline-recommended standard for performing intubations prehospitally. First pass success for SAD was high. This adds support for current Dutch ambulance guidelines recommending an SAD for primary choice of advanced airway by EMS during CPR. However, improvement of intubation techniques and skills remain a necessity for selected OHCA patients.

## Highlights

• Changes in OHCA airway management protocols resulted in more SAD and less ETT placements by EMS.
• ETT first pass success rates increased to 68%, SAD first pass success was steady and averaged at 93%
• For patients needing an ETT, efforts should be made in improving first pass success rate.

## Introduction

Cardiopulmonary resuscitation (CPR) consists of two main components: oxygenation of blood and transporting oxygenated blood to vital organs, preventing ischemia. An open airway is the first step in facilitating oxygenation.^1^ After arrival on scene of an out-of-hospital cardiac arrest (OHCA), Emergency Medical Service (EMS) providers start chest compressions and bag-valve-mask (BVM) ventilation. Ventilation with a BVM can be started rapidly, but requires constant attention. Even in experienced hands, opening the airway and ventilating the patient can be challenging.^2^, ^3^

After BVM ventilation, advanced airway management can be provided. Endotracheal tube (ETT) placement has long been regarded as “gold standard” for airway management during CPR, as it provides the patient with a secured airway and reduces stomach inflation and aspiration. However, aspiration usually occurs before EMS providers arrive, and might happen during, and possibly even after intubation, which limits its use for prevention of aspiration.^2^, ^4^ Intubation first pass success is associated with a higher likelihood of return of spontaneous circulation (ROSC).^5^ Endotracheal intubation by providers with limited experience leads to delay in providing adequate chest compressions and treatment, and could result in long apnea intervals associated with worse survival.^6^, ^7^ The supgraglottic airway device (SAD) also allows proper ventilation in a vast number of patients, while skills required for SAD placement are easier to learn and maintain.^8^, ^9^, ^10^, ^11^

Several studies comparing SAD and ETT placement found similar, or sometimes, better results regarding survival in favor of SADs, resulting in a protocol shift towards SAD use in both international and national EMS protocols for advanced airway management during OHCA.^4^, ^12^, ^13^, ^14^, ^15^, ^16^, ^17^ This potentially alters the experience and, thus, success with advanced airway devices.^18^, ^19^, ^20^, ^21^, ^22^, ^23^, ^24^, ^25^ Trends towards a declined ETT and inclined SAD use are seen in the USA, but it’s influence on placement success is unknown.^26^, ^27^

The aim of this study is to assess how airway management is performed by Dutch EMS during CPR over time. If we know what the effects are of protocol changes on trends in airway management strategies and performance, we can determine the potential need for interventions such as airway management training, other strategies or devices.

## Methods

### Setting

In the Netherlands, two advanced life support (ALS) level ambulances, including one ambulance nurse and one driver, are dispatched for each (suspected) OHCA. Ambulance nurses are trained to perform airway management including BVM ventilations, SAD placement, endotracheal intubation and emergency front-of-neck access (eFONA). Additionally, volunteer community responders are alerted and first responders are dispatched to facilitate early Basic Life Support (BLS) and defibrillation.^28^, ^29^

For specific OHCA patients, Helicopter Emergency Medical Services (HEMS) is also dispatched.^30^ These include patients with suspected airway problems, pregnant patients, pediatric OHCA (<18 years), or patients with trauma, electrocution or drowning as cause of arrest. HEMS is also deployed when a first ambulance is not expected to be on scene within ten minutes. Lastly, HEMS in the ARREST (Amsterdam Resuscitation Studies) study region have temporarily been deployed since October 2022 for OHCA patients between 18 and 50 years of age with a witnessed arrest, as part of the ON-SCENE study.^31^

All ambulance nurses follow national ambulance guidelines based on the Dutch and European Resuscitation Council (ERC) Guidelines.^32^ In the national ambulance guideline of 2014, the use of SAD was introduced, and the choice of advanced airway was at the discretion of the provider or regional ambulance service.^15^ The included ambulance services did not use advanced airway adjuncts (such as video laryngoscopy or gum elastic bougie) during the study period. During the COVID-19 pandemic, guidelines were temporarily altered: from April-June 2020, EMS were advised not to provide BVM ventilations and directly insert an SAD (preferred choice) or ETT (second choice).^33^ From June 2020, this only applied to patients suspected for COVID-19, and after September 2021, all COVID-modifications were dismissed.^34^, ^35^ The strategy of direct SAD placement, omitting BVM ventilations, is not yet described as an option in resuscitation guidelines outside of the COVID-19 OHCA context. In the 2023 guidelines, effective from May 1st 2023 on, it was stated that using an ETT was only recommended if SAD has not been effective or achievable.^17^ This was an interpretation of the 2021 ERC Guidelines, which advised that only systems with a high intubation success rate of 95% after 2 attempts should use ETI.^32^

### Study design and data source

This is an observational study, based on data from the ARREST registry: an ongoing prospective database including all patients with an (suspected) OHCA since 2005, in the province of North-Holland.^36^ This region covers approximately 2.7 million inhabitants.^37^ ARREST registers Utstein guideline-recommended data for OHCA, including the following data sources: dispatch centers, the national volunteer responder system, automated external defibrillator (AED) recordings, EMS manual defibrillators, EMS run reports, EMS questionnaires, and hospitals.^36^, ^38^ Informed consent is obtained in survivors to include the full dataset. When consent is not given or cannot be obtained, use of a limited anonymous dataset is still granted. For deceased patients, informed consent is waived for the full dataset.

Systematic collection of airway related variables started in 2019, data on HEMS-dispatch and arrival were recorded from October 2022. For patients included before October 2022, ARREST data were matched with data from the HEMS-database to check retrospectively for HEMS-presence during CPR. For patients in this time period with an anonymous dataset (±15% of patients), matching the databases was not possible. These ±1034 patients were not excluded from the analysis due to the low expected amount (±66 patients) that should have been excluded due to HEMS-presence during CPR. Airway related variables, reported by ambulance nurses in the EMS questionnaire, include order of successfully placed airway devices, first-pass success of ETT- and SAD placement, and type of definitive airway device during cardiac arrest. First pass success was defined as the ability to ventilate the patient adequately after the first placement attempt. If successful placement occured after the first unsuccesfull attempt, it was included in the order of successfully placed airway devices. The definitive airway device was defined as the device in place at prehospital termination of CPR, or at emergency department arrival during CPR.

The Medical Research Involving Human Subjects Act did not apply to this study as reviewed and stated by the medical ethics review board of the Amsterdam UMC (number W17_089).

### Patient selection

All consecutive adult (≥18 years) OHCA patients who had resuscitation attempted by EMS were included. Resuscitation attempted by EMS was defined as having received a two minute-cycle of CPR at least, or having received at least one shock by an EMS defibrillator. Cases where HEMS were primarily dispatched due to the cardiac arrest etiology or where they were dispatched secondarily and registered to be present on scene, and could possibly have performed the airway maneuvers, were excluded.

### Objectives

The primary outcome was the percentage of adult OHCA cases in which BVM, SAD, ETT or eFONA were used as definitive airway device during CPR, classified by year, in the period of 2019 to 2023. Secondarily, we analyzed the difference in intra-arrest first pass success rate of SADs and ETTs, classified by year. This study was not designed to relate these airway management strategies to patient outcomes.

### Sample size calculation

An a priori sample size calculation was not performed as the sample size was fixed by the available number of patients in the database during the study period. Therefore, we calculated if we would be able to detect a clinically relevant difference of a change of 5% in the use of a particular airway device during this 5-year study.^39^ The available number of 5222 analyzed patients provide 80% power to detect a 5.6% change (either in- or decrease, i.e., two-sided hypothesis) at a 0.05 alpha level.

### Statistical analysis

Calculating the probability to detect a clinically relevant difference was performed with Stata/BE 17.0 (StataCorp LLC). Statistical analysis was performed with SPSS® (version 28.0, IBM® SPSS®, Chicago, IL). Baseline characteristics and outcomes were described as percentages for categorical data, mean and standard deviation or median and interquartile range were used for continuous data. Chi-square test for trends was used to test for difference between years, including the primary outcome. Proportions with 95% confidence intervals (CI) were calculated for the primary outcome. To test for significant differences in categorical variables between airway groups chi-square tests were used. When expected cell counts of 5 or below were found, Fisher’s exact tests, for two groups, or Fisher-Freeman-Halton Exact tests, for more than two groups, were used. For comparisons of continuous variables between airway groups, Kruskal-Wallis tests were used.

Due to the small number (4) of patients with eFONAs, these patients were post-hoc excluded from the analysis to reduce the risk of re-identification.

## Results

**Fig. 1:**
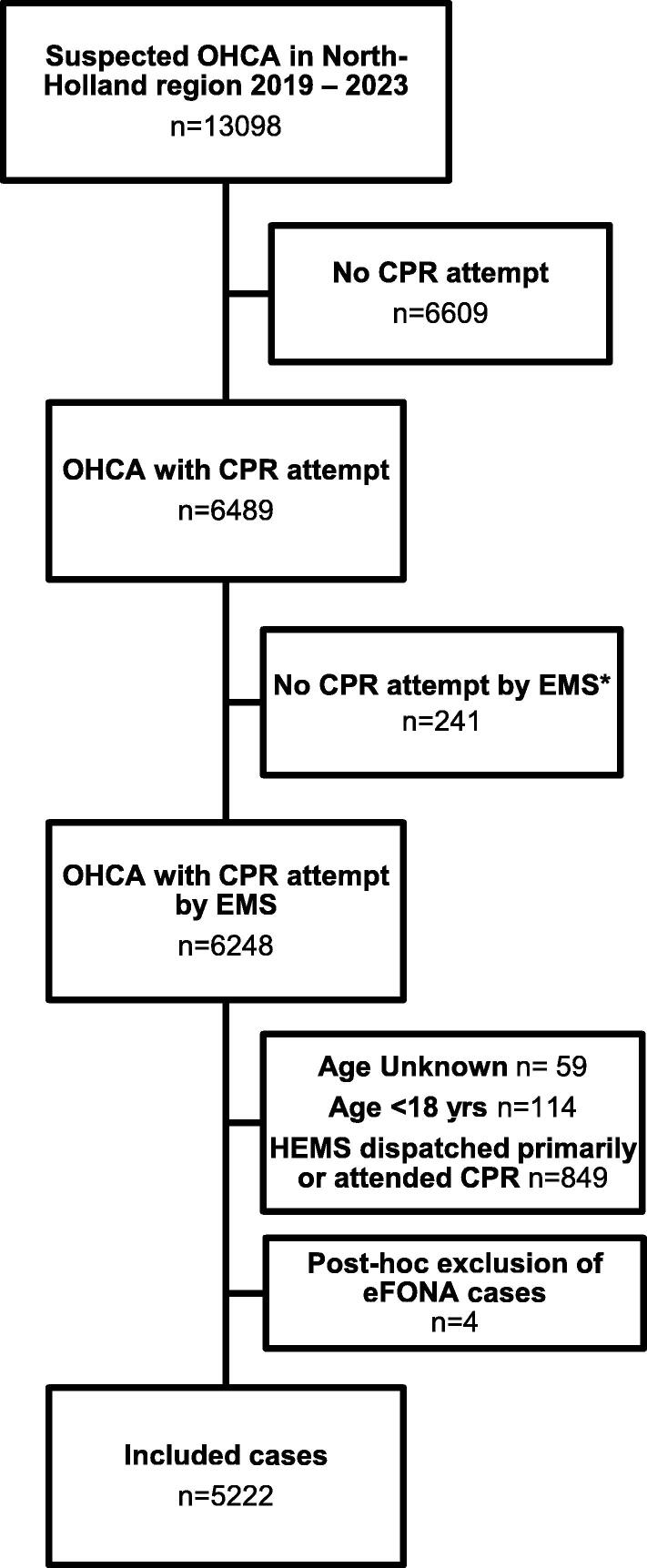
Flow diagram of inclusion. CPR: cardiopulmonary resuscitation, eFONA: emergency front-of-neck access, EMS: Emergency Medical Services, HEMS: Helicopter Emergency Medical Services, OHCA: out-of-hospital cardiac arrest. *ROSC due to successful defibrillation by AED before EMS arrival.

Of 13,098 cases suspected for OHCA in 2019–2023, 6248 cases underwent a resuscitation attempt by EMS. After exclusion of children, cases where HEMS were primarily dispatched or present secondarily, and those with eFONAs, 5222 cases remained (Fig. 1). The majority of patients (59%, 95% CI: 57–60%) had an SAD as definitive airway, 14% (95% CI: 13–15%) a BVM, and 21% (95% CI: 19–22%) had an ETT at the end of CPR by EMS (Table 1).

**Table 1:** Baseline characteristics of included cases stratified by definitive airway device.

	BVM	SAD	ETT	Unknown	Total	Statistics
Adult OHCA with CPR attempt, *n* (%; 95% CI)	711 (14; 13–15)	3062 (59; 57–60)	1072 (21; 19–22)	377 (7; 7–8)	**5222**	
Male sex, *n* (%)	521 (73)	2130 (70)	742 (69)	267 (71)	**3660 (70)**	*p* = 0.225
Missing	0	1 (0)	0	1 (0)	**2 (0)**	
Age in years, Median (IQR)	67 (55 – 76)	70 (60–78)	72 (62–80)	71 (60–79)	**70 (60**–**78)**	*p* < 0.001
First rhythm, *n* (%)						*p* < 0.001
Shockable (VF/VT)	301 (42)	873 (29)	340 (32)	102 (27)	**1616 (31)**	
PEA	116 (16)	978 (32)	329 (31)	90 (24)	**1513 (29)**	
Asystole	119 (17)	910 (30)	291 (27)	104 (28)	**1424 (27)**	
EMS Witnessed*	149 (21)	226 (7)	71 (7)	55 (15)	**501 (10)**	
Unknown**	26 (4)	75 (2)	41 (4)	26 (7)	**168 (3)**	
Cause of arrest, *n* (%)						*p* = 0.347
Medical	705 (99)	3032 (99)	1063 (99)	370 (98)	**5170 (99)**	
Drug overdose	6 (1)	30 (1)	9 (1)	7 (2)	**52 (1)**	
Arrest witness, *n* (%)						*p* < 0.001
Bystander	372 (52)	1773 (58)	676 (63)	161 (43)	**2982 (57)**	
EMS	151 (21)	229 (8)	71 (7)	57 (15)	**508 (10)**	
None	178 (25)	1033 (34)	312 (29)	128 (34)	**1651 (32)**	
Missing	10 (1)	27 (1)	13 (1)	31 (8)	**81 (2)**	
BLS before EMS arrival, *n* (%)	564 (79)	2470 (81)	865 (81)	291 (77)	**4190 (80)**	*p* = 0.812
Missing	3 (0)	16 (1)	5 (1)	21 (6)	**45 (1)**	
AED before ambulance arrival, *n* (%)	382 (54)	1690 (55)	609 (57)	200 (53)	**2881 (55)**	*p* = 0.540
Missing	1 (0)	14 (1)	7 (1)	11 (3)	**33 (1)**	
AED/ EMS defibrillation, *n* (%)	459 (65)	1300 (43)	501 (47)	175 (46)	**2435 (47)**	*p* < 0.001
Missing	1 (0)	10 (0)	3 (0)	17 (5)	**31 (1)**	
ROSC before transportation, *n* (%)						*p* < 0.001
Yes	425 (60)	867 (28)	360 (34)	94 (25)	**1746 (33)**	
No	32 (5)	700 (23)	224 (21)	41 (11)	**997 (19)**	
N/A***	251 (35)	1474 (48)	479 (45)	217 (58)	**2421 (46)**	
Missing	3 (0)	21 (1)	9 (1)	25 (7)	**58 (1)**	
Result prehospital CPR, *n* (%)						*p* < 0.001
ROSC	394 (55)	622 (20)	287 (27)	81 (22)	**1384 (27)**	
Transport to hospital continuing CPR	87 (12)	946 (31)	290 (27)	68 (18)	**1391 (27)**	
Deceased	227 (32)	1479 (48)	486 (45)	205 (54)	**2397 (46)**	
Missing	3 (0)	15 (1)	9 (1)	23 (6)	**50 (1)**	
Alert to start CPR by EMS mm:ss, Median (IQR)	11:26 (9:06–13:53)	11:25 (9:21–13:54)	10:49 (8:47–13:25)	11:24 (9:29–13:44)	**11:16 (9:14**–**13:47)**	*p* < 0.001
Missing	159 (22)	346 (11)	133 (12)	107 (28)	**745 (14)**	
Alert to ROSC mm:ss, Median (IQR)	15:36 (12:09–22:32)	23:30 (18:36–29:10)	23:30 (18:24–28:38)	22:32 (13:52–33:59)	**22:26 (16:49**–**28:27)**	*p* < 0.001
Missing or no ROSC	401 (56)	2140 (70)	712 (66)	288 (76)	**3541 (68)**	
Start CPR by EMS to ROSC mm:ss, Median (IQR)	4:09 (1:55–10:31)	11:56 (7:55–17:14)	12:06 (8:21–17:30)	9:03 (3:25–18:03)	**10:54 (6:03**–**16:45)**	*p* < 0.001
Missing or no ROSC	405 (57)	2140 (70)	712 (66)	291 (77)	**3548 (68)**	

AED: automated external defibrillator, BLS: basic life support, BVM: bag-valve-mask, CPR: cardiopulmonary resuscitation, EMS: emergency medical services, ETT: endotracheal tube, IQR: interquartile range, SAD: supraglottic airway device, OHCA: out-of-hospital cardiac arrest, ROSC: return of spontaneous circulation, VF: ventricular fibrillation, VT: ventricular tachycardia.

* For EMS-witnessed cases, the first rhythm on the ambulance monitor is not the first recorded rhythm during cardiac arrest.

** The first monitored rhythm was either missing or difficult to classify for cases with an unknown first monitored rhythm.

*** N/A = not applicable. Cases where ROSC before transportation was not applicable were patients with an EMS-witnessed arrest and/or those that EMS decided not to transport.

Patients with a BVM as the definitive airway were younger than those with an advanced airway (67 vs. 71 years), and more often experienced an EMS-witnessed arrest (21% vs. 7%), had a shockable first rhythm (42% vs. 29%) and achieved ROSC (55% vs. 22%, Table 1). The median time interval from start of CPR by EMS to ROSC was 4:09 (IQR 1:55–10:31) in the BVM-group versus 11:56 (IQR 8:03–17:19) in patients with an advanced airway. In the group with a BVM at termination of CPR, an attempt at an advanced airway was made in 120 cases (17%; attempt at SAD occurred in 13%, and at ETT in 11% of patients), and no attempt was made in 568 cases (80%). For 23 cases (3%), this was unknown (Table 2).

**Table 2:** Airway characteristics of included cases stratified by definitive airway device.

	BVM	SAD	ETT	Unknown	Total	Statistics
Adult OHCA with CPR attempt, *n* (%; 95% CI)	711 (14; 13–15)	3062 (59; 57–60)	1072 (21; 19–22)	377 (7; 7–8)	5222	
SAD attempted, *n* (%)	90 (13)	3062 (100)	112 (10)	7 (2)	3271 (63)	*p* < 0.001
Missing	23 (3)	0	40 (4)	365 (97)	428 (8)	
First pass success SAD, *n* (% of attempted)	1 (1)	3002 (98)	44 (39)	4 (57)	3051 (93)	*p* < 0.001
Tube attempted, *n* (%)	75 (11)	498 (16)	1072 (100)	11 (3)	1656 (32)	*p* < 0.001
Missing	24 (3)	180 (6)	0	358 (95)	562 (11)	
First pass success tube, *n* (% of attempted)	0 (0)	3 (1)	1017 (95)	4 (36)	1024 (62)	*p* < 0.001
Order of successful airway devices, *n* (%)						*p* < 0.001
BVM	708 (100)	0	0	0	708 (14)	
BVM + SAD	0	1996 (65)	0	0	1996 (38)	
BVM + ETT	0	0	871 (81)	0	871 (17)	
BVM + SAD + ETT	0	0	39 (4)	0	39 (1)	
BVM + ETT + SAD	0	2 (0)	0	0	2 (0)	
SAD	0	1003 (33)	0	0	1003 (19)	
SAD + ETT	0	0	5 (1)	0	5 (0)	
ETT	0	0	38 (4)	0	38 (1)	
ETT + SAD	0	1 (0)	0	0	1 (0)	
Missing	3 (0)	60 (2)	119 (11)	377 (100)	559 (11)	

BVM: bag-valve-mask, CPR: cardiopulmonary resuscitation, ETT: endotracheal tube, SAD: supraglottic airway device, OHCA: out-of-hospital cardiac arrest.

**Fig. 2:**
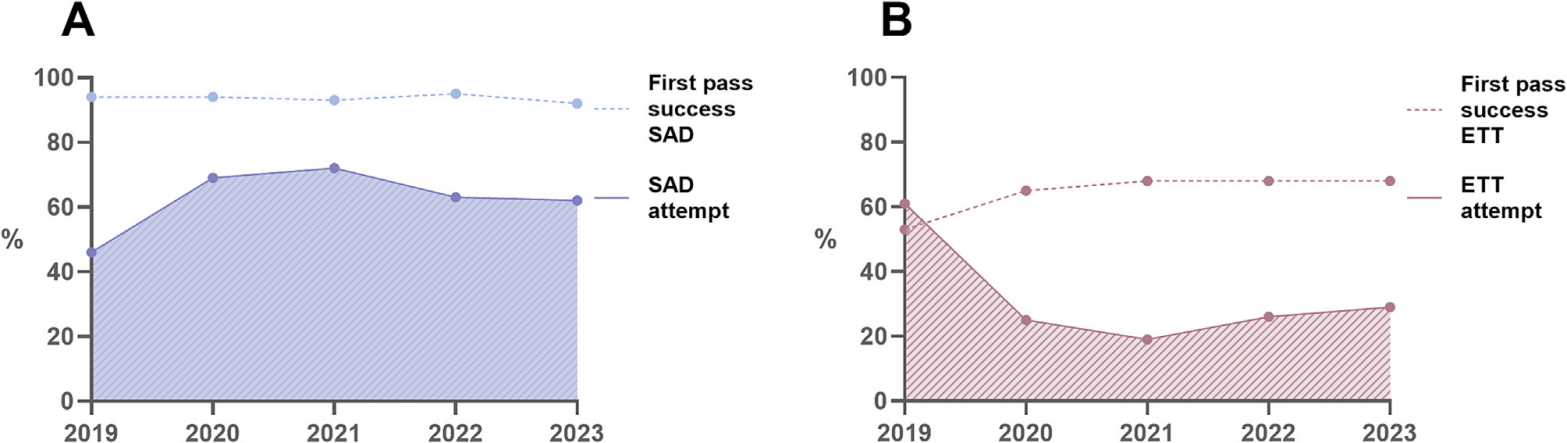
A. Percentage of patients with SAD attempt and SAD first pass success over the years. B. Percentage of patients with ETT attempt and ETT first pass success over the years. ETT: endotracheal tube, SAD: supraglottic airway device.

A witnessed arrest (65% vs. 70%), defibrillation by AED or EMS defibrillator (43% vs. 47%), and ROSC before transportation (28% vs. 34%) or transport to hospital with ROSC (20% vs. 27%) occurred less often in patients with an SAD than in those with an ETT at the end of CPR by EMS. Ongoing CPR during transportation was more prevalent in patients with an SAD than with an ETT (31% vs. 27%). Supplementary table A shows baseline characteristics of OHCA patients over the years. SAD placement was mostly successful on first attempt (93%), which was consistent over the years (Table 3, Fig. 2A). In 44 patients, intubation was performed and succeeded after successful placement of an SAD (1% of patients with a successfully placed SAD). First pass success of intubation was 62% on average, though a trend was seen towards a higher first pass success (53% in 2019; 68% in 2023), even though the proportion of cases in which intubation was attempted decreased over the years (61% in 2019, 29% in 2023; Fig. 2B, supplementary Fig. A).

**Table 3:** Airway characteristics of included cases stratified by year of arrest.

	2019	2020	2021	2022	2023	Total	Statistics
Adult OHCA with CPR attempt, *n* (%)	1011 (19)	1000 (19)	1111 (21)	1093 (21)	1007 (19)	5222	
SAD attempted, *n* (%)	463 (46)	694 (69)	801 (72)	685 (63)	627 (62)	3270 (63)	*p* < 0.001
Missing	103 (10)	61 (6)	95 (9)	86 (8)	84 (8)	429 (8)	
First pass success SAD, *n* (% of attempted)	436 (94)	650 (94)	740 (92)	647 (95)	578 (92)	3051 (93)	*p* = 0.378 (X20.378 (X
Tube attempted, *n* (%)	617 (61)	249 (25)	211 (19)	288 (26)	291 (29)	1656 (32)	*p* < 0.001
Missing	186 (18)	107 (11)	99 (9)	90 (8)	80 (8)	562 (11)	
First pass success tube, *n* (% of attempted)	325 (53)	161 (65)	144 (68)	197 (68)	197 (68)	1024 (62)	*p* < 0.001
Definitive device, *n* (%; 95% CI)							*p* < 0.001
BVM	169 (17; 15–19)	131 (13; 11–15)	121 (11; 9–13)	158 (15; 13–17)	132 (13; 11–15)	711 (14; 13–15)	
SAD	434 (43; 40–46)	651 (65; 62–68)	752 (68; 65–70)	644 (59; 56–62)	581 (58; 55–61)	3062 (59; 57–60)	
ETT	329 (33; 30–36)	167 (17; 15–19)	156 (14; 12–16)	211 (19; 17–22)	209 (21; 18–23)	1072 (21; 19–22)	
Unknown (missing)	79 (8; 6–10)	51 (5; 4–7)	82 (7; 6–9)	80 (7; 6–9)	85 (8; 7–10)	377 (7; 7–8)	
Order of successful airway devices, *n* (%)							*p* = 0.005
BVM	169 (17)	131 (13)	121 (11)	157 (14)	130 (13)	708 (14)	
BVM + SAD	432 (43)	257 (26)	244 (22)	530 (49)	533 (53)	1996 (38)	
BVM + ETT	325 (32)	119 (12)	71 (6)	161 (15)	195 (19)	871 (17)	
BVM + SAD + ETT	3 (0)	7 (1)	5 (1)	15 (1)	9 (1)	39 (1)	
BVM + ETT + SAD	0 (0)	0 (0)	1 (0)	1 (0)	0 (0)	2 (0)	
SAD	0 (0)	370 (37)	488 (44)	99 (9)	46 (5)	1003 (19)	
SAD + ETT	0 (0)	3 (0)	1 (0)	0 (0)	1 (0)	5 (0)	
ETT	0 (0)	15 (2)	10 (1)	12 (1)	1 (0)	38 (1)	
ETT + SAD	0 (0)	0 (0)	1 (0)	0 (0)	0 (0)	1 (0)	
Missing	82 (8)	98 (10)	169 (15)	118 (11)	92 (9)	559 (11)	

BVM: bag-valve-mask, CPR: cardiopulmonary resuscitation, ETT: endotracheal tube, SAD: supraglottic airway device, OHCA: out-of-hospital cardiac arrest.

**Fig. 3:**
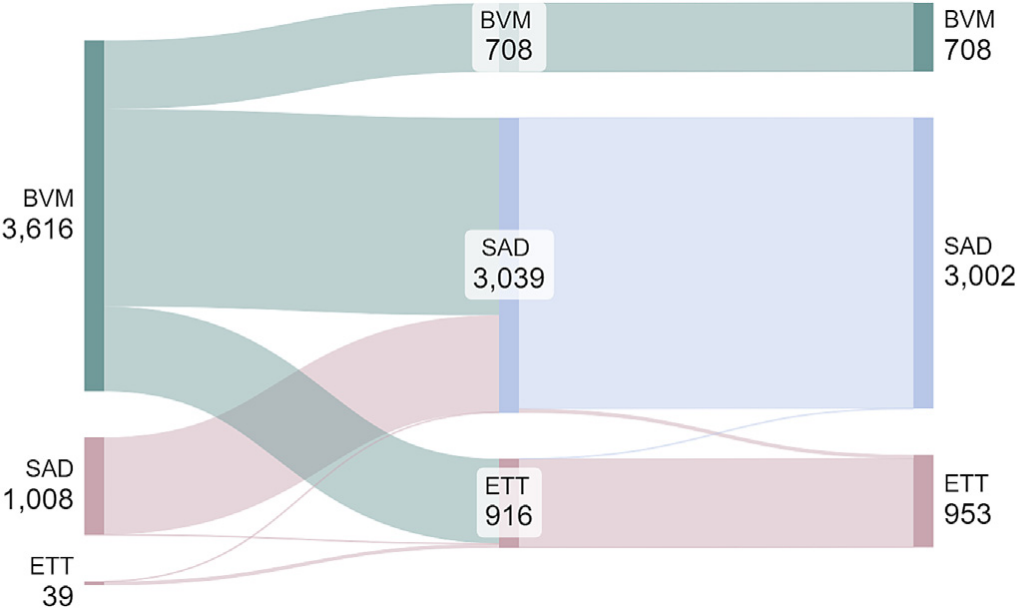
Sankey diagram of successful airway management order. BVM: bag-valve-mask, ETT: endotracheal tube, SAD: supraglottic airway device.

The most used successful airway management strategy in OHCA patients was BVM ventilation followed by SAD placement (38%). Over the years with airway-related COVID-restrictions, direct SAD placement seemed to have partly replaced all types of strategies starting with BVM-ventilations. This strategy was still observed after withdrawal of those restrictions. Advanced airway management by direct ETT placement during COVID-restrictions was not a regularly performed strategy. The order of successful airway management strategies is displayed in a Sankey diagram (Fig. 3). One patient in the BVM-group had a successfully placed SAD, after which an unsuccessful attempt at intubation followed, and BVM was used afterwards without another advanced airway attempt. Of patients with an initial successfully placed ETT, three patients received a (successfully placed) SAD afterwards. For one patient, the reason for switching back to SAD was assumed esophageal placement because of the low end-tidal CO_2_-level, which remained low after SAD placement. No additional information was provided for the other two cases.

## Discussion

From 2019 to 2023, use of an ETT as the definitive airway device decreased from 33% (95% CI: 30–36%) to 21% (95% CI: 18–23%). The opposite was seen for SADs, with an increase from 43% (95% CI: 40–46%) to 58% (95% CI: 55–61%).

Over the course of five years, ETT first pass success improved from 53% in 2019 to 68% in 2023, though less intubations were performed. Over the same time period, SAD first pass success remained consistently high with 93% on average. Unchanged intubation success rates during the COVID-19 pandemic, described by Armour et al., similarly demonstrated that the significantly reduced proportion of cases with ETT attempts did not harm intubation success.^40^ The outbreak of the COVID-19 pandemic caused a decreased use of ETTs, and increased use of SADs, as was seen in a meta-analysis by Kim et al. evaluating prehospital airway management during the pandemic.^41^ Even after returning to pre-COVID guidelines, the increased use of SADs sustained in this cohort. It can be argued that, in our study, because of the imposed primary SAD placement, individually improved experience with SADs led to a change in preference of primary airway device by these EMS nurses. After the pandemic, a selection may have occurred in the intubation complexity level of the patients and of EMS providers with higher intubation skill level, resulting in the improved ETT first pass success overall.

Interestingly, while the most recent ambulance protocol primarily mandates SAD insertion before attempting endotracheal intubation, only 10% of patients with an ETT as definitive device underwent an attempt at SAD placement. Although the proportion of patients undergoing an SAD attempt increased from 46% in 2019 to 62% in 2023, no attempt was made in at least 29% of patients in 2023, even after the recommendation for a primary SAD attempt came into effect in May 2023. The motivation for choice of airway device was not registered in this study. Therefore, it is possible that an SAD was deemed unsuitable for these patients by EMS. Aspiration could be the reason; the incidence of aspiration during OHCA is maximally estimated at 32%, and the presence of oral fluids was the main reason to deviate from SAD-first protocol in the AIRWAYS-2 trial.^4^, ^13^, ^42^, ^43^, ^44^, ^45^ In case of regurgitation, paramedics are approximately five times more likely to place an ETT over SAD. However, although EMS could be trained to prevent or manage aspiration by intubation instead of SAD placement, the incidence of regurgitation and aspiration during or after advanced airway management is not significantly different between SAD or ETT placement during OHCA.^4^ It could therefore be argued that a primary SAD attempt, as described in current guidelines, could also have been a reasonable advanced airway management strategy for a part of these patients. In an earlier study by Berdowski, implementation of (national) resuscitation guidelines took 1.5 years, which promises improvement of guideline adherence.^46^

There are several limitations to this study. First, airway management strategies and their success rates were self-reported by ambulance nurses. Bias, most probably resulting in an increased first pass success reported, could have occurred. The heterogeneous definition of first pass success makes it difficult for EMS to interpret, and could have attributed to this bias.^47^ Next, this study only describes the process of airway management by ambulance nurses, but does not evaluate why certain choices were made, nor the association with patient outcomes. This limits the possibility of making assumptions about the validity of choices on airway management strategies made by ambulance nurses. However, the results could be used to determine if and where support to ambulance nurses should be offered by providing airway management training, or developing other strategies or devices.

Although the results of this study might support stimulating EMS to primarily attempt SAD placement, for some patients, such as those who had an ultimately failed attempt at SAD placement (±3% of patients in this cohort) or who underwent an ETT attempt after initial successful SAD placement (1% of patients in this cohort), advanced airway management by intubation would remain necessary. For this group, improving first pass success for intubations by EMS is needed, as more than one-third of patients was not successfully intubated at first attempt. First pass success for ETT placement in OHCA by EMS ranges from 51% to 90%, depending on the definition of first pass success, setting, and type and training of providers.^3^, ^4^, ^13^, ^23^, ^48^, ^49^, ^50^ The first pass success of ETI in this cohort is within that range, but not considered “high” when interpreting the 2021 ERC guidelines. Adequate intubation training remains vital, and, additionally, implementation of a bougie and/or videolaryngoscopy might be considered.^51^, ^52^ Videolaryngoscopy has been shown to improve first pass success for less experienced providers, and has been associated with improved survival.^53^, ^54^, ^55^, ^56^, ^57^

We encourage future randomized trials comparing the impact of videolaryngoscopy with direct laryngoscopy on first pass success and outcomes in these patients. Furthermore, the impact of a direct SAD placement versus BVM first strategy on ventilation performance and outcomes deserves further attention, as this study shows that this is a strategy practiced by EMS. Studies on airway management should ideally include ventilation metrics, to properly evaluate the effects on ventilation, because, after all, airway management is a means to achieve adequate oxygenation and ventilation, not an end in itself.^58^, ^59^

## Conclusion

In recent years, the main advanced airway device used at OHCAs shifted from ETTs towards SADs. First pass success for SADs was high, and the proportion of patients with attempts at intubation following attempt(s) at SAD placement was low. Moreover, in this study, a counter-intuitive improvement of ETT first pass success was seen following the decrease in the proportion of patients in which an attempt at intubation was undertaken. This study therefore shows that a primary SAD placement strategy for EMS remains reasonable, even after aiway management practices have changed over time. However, intubation is still required for some patients, and improvement strategies are needed to increase ETT first pass success for EMS.

## CRediT authorship contribution statement

**Lotte C. Doeleman:** Writing – review & editing, Writing – original draft, Visualization, Validation, Software, Methodology, Investigation, Formal analysis, Data curation, Conceptualization. **Julian F.F. de Jong:** Writing – review & editing, Writing – original draft, Visualization, Validation, Software, Methodology, Investigation, Formal analysis, Data curation. **Vera G.M. van Eeden:** Writing – review & editing, Resources, Investigation, Formal analysis, Data curation. **Patrick Schober:** Writing – review & editing, Methodology, Investigation, Formal analysis. **Markus W. Hollmann:** Writing – review & editing, Supervision, Project administration. **Hans van Schuppen:** Writing – review & editing, Writing – original draft, Validation, Supervision, Resources, Project administration, Methodology, Investigation, Conceptualization.

## Funding

The ARREST registry is maintained by an unconditional grant of Stryker Emergency Care, Redmond, WA. This study has not received specific funding.

## Declaration of competing interest

The authors declare the following financial interests/personal relationships which may be considered as potential competing interests: ‘LD and HvS report a grant to their institution from Stryker Emergency Care. JdJ and VE report no conflict of interest. PS reports a grant from Health Holland, outside the scope of this study. MWH reports grants to his institution from ZonMW and ESAIC, and consulting fees paid to his institution from IDD Pharma, Medical Developments and PAION, all outside the scope of this study.’.
