# Supplementary material for: Airway management by ambulance nurses during out-of-hospital cardiac arrest
Source: Resusc Plus. 2025 Jun 8;25:100999. doi: 10.1016/j.resplu.2025.100999 (PMC12214125; doi:10.1016/j.resplu.2025.100999)
Supplement: Supplementary Data 1 [file mmc1.docx]

Appendix: Supplementary material

| **Supplementary table A – Baseline characteristics of included cases stratified by year of arrest** | | | | | | | |
| --- | --- | --- | --- | --- | --- | --- | --- |
|  | **2019** | **2020** | **2021** | **2022** | **2023** | **Total** | **Statistics** |
| **Adult OHCA with CPR attempt, n (%)**  **n (% of total)** | 1011 (19) | 1000 (19) | 1111 (21) | 1093 (21) | 1007 (19) | **5222 (100)** |  |
| **Male sex, n (%)** | 722 (71) | 709 (71) | 759 (68) | 772 (71) | 698 (69) | **3660 (70)** | p = 0.319 |
| Missing | 1 (0) | 0 | 1 (0) | 0 | 0 | **2 (0)** |  |
| **Age in years, Median (IQR)** | 70 (58 – 79) | 69 (59 – 78) | 69 (59 – 78) | 70 (59 – 78) | 72 (62 – 79) | **70 (60 – 78)** | p < 0.001 |
| **First rhythm, n (%)** |  |  |  |  |  |  | p = 0.943 |
| Shockable (VF/VT) | 324 (32) | 317 (32) | 332 (30) | 337 (31) | 306 (30) | **1616 (31)** |  |
| PEA | 230 (23) | 208 (21) | 364 (33) | 440 (40) | 271 (27) | **1513 (29)** |  |
| Asystole | 309 (31) | 347 (35) | 293 (26) | 183 (17) | 292 (29) | **1424 (27)** |  |
| EMS Witnessed* | 67 (7) | 105 (11) | 101 (9) | 116 (11) | 112 (11) | **501 (10)** |  |
| Unknown** | 81 (8) | 23 (2) | 21 (2) | 17 (2) | 26 (3) | **168 (3)** |  |
| **Cause of arrest, n (%)** |  |  |  |  |  |  | p = 0.830 |
| Medical | 1006 (100) | 984 (98) | 1101 (99) | 1080 (99) | 999 (99) | **5170 (99)** |  |
| Drug overdose | 5 (1) | 16 (2) | 10 (1) | 13 (1) | 8 (1) | **52 (1)** |  |
| **Arrest witness, n (%)** |  |  |  |  |  |  | p < 0.477 |
| Bystander | 635 (63) | 538 (54) | 631 (57) | 633 (58) | 545 (54) | **2982 (57)** |  |
| EMS | 72 (7) | 105 (11) | 101 (9) | 117 (11) | 113 (11) | **508 (10)** |  |
| Unwitnessed | 284 (28) | 344 (34) | 364 (33) | 326 (30) | 333 (33) | **1651 (32)** |  |
| Missing | 20 (2) | 13 (1) | 15 (1) | 17 (2) | 16 (2) | **81 (2)** |  |
| **BLS before EMS arrival, n (%)** | 775 (77) | 747 (75) | 847 (76) | 949 (87) | 872 (87) | **4190 (80)** | p < 0.001 |
| Missing | 10 (1) | 6 (1) | 19 (2) | 6 (1) | 4 (0) | **45 (1)** |  |
| **AED before ambulance arrival, n (%)** | 565 (56) | 534 (53) | 615 (55) | 613 (56) | 554 (55) | **2881 (55)** | p = 0.759 |
| Missing | 0 | 8 (1) | 12 (1) | 8 (1) | 5 (1) | **33 (1)** |  |
| **AED/ EMS defibrillation, n (%)** | 482 (48) | 492 (49) | 494 (45) | 501 (46) | 466 (46) | **2435 (47)** | p = 0.169 |
| Missing | 9 (1) | 9 (1) | 5 (1) | 2 (0) | 6 (1) | **31 (1)** |  |
| **ROSC before transportation, n (%)** |  |  |  |  |  |  | p < 0.001 |
| Yes | 382 (38) | 340 (34) | 365 (33) | 344 (32) | 315 (31) | **1746 (33)** |  |
| No | 210 (21) | 177 (18) | 199 (18) | 213 (20) | 198 (20) | **997 (19)** |  |
| N/A *** | 396 (39) | 472 (47) | 537 (48) | 531 (49) | 485 (48) | **2421 (46)** |  |
| Missing | 23 (2) | 11 (1) | 10 (1) | 5 (1) | 9 (1) | **58 (1)** |  |
| **Result prehospital CPR, n (%)** |  |  |  |  |  |  | p = 0.016 |
| ROSC | 314 (31) | 281 (28) | 267 (24) | 280 (26) | 242 (24) | **1384 (27)** |  |
| Transport to hospital continuing CPR | 238 (24) | 244 (24) | 312 (28) | 306 (28) | 291 (29) | **1391 (27)** |  |
| Deceased | 443 (44) | 467 (47) | 522 (47) | 501 (46) | 464 (46) | **2397 (46)** |  |
| Missing | 16 (2) | 8 (1) | 10 (1) | 6 (1) | 10 (1) | **50 (1)** |  |
| **Alert to start CPR by EMS mm:ss, Median (IQR)** | 11:15 (8:59 – 13:52) | 11:18 (9:17 – 13:50) | 11:21 (9:22 – 13:42) | 11:06 (9:15 – 13:56) | 11:25 (9:08 – 13:39) | **11:16 (9:14 – 13:47)** | p = 0.935 |
| Missing | 174 (17) | 172 (17) | 153 (14) | 122 (11) | 124 (12) | **745 (14)** |  |
| **Alert to ROSC mm:ss, Median (IQR)** | 21:24 (16:12 – 27:20) | 22:47 (17:12 – 28:23) | 22:35 (17:41 – 28:24) | 23:21 (17:24 – 29:58) | 21:28 (15:40 – 28:54) | **22:26 (16:49 – 28:27)** | p = 0.136 |
| Missing or no ROSC | 696 (69) | 678 (68) | 737 (66) | 744 (68) | 686 (68) | **3541 (68)** |  |
| **Start CPR by EMS to ROSC mm:ss, Median (IQR)** | 10:20 (6:21 – 16:29) | 10:57 (6:12 – 16:56) | 11:00 (6:01 – 16:06) | 11:42 (6:34 – 16:35) | 10:33 (5:21 – 17:40) | **10:54 (6:03 – 16:45)** | p = 0.673 |
| Missing or no ROSC | 700 (69) | 678 (68) | 737 (66) | 746 (68) | 687 (68) | **3548 (68)** |  |

*For EMS-witnessed cases, the first rhythm on the ambulance monitor is not the first recorded rhythm during cardiac arrest.

** The first monitored rhythm was either missing or difficult to classify for cases with an unknown first monitored rhythm

*** N/A = not applicable. Cases where ROSC before transportation was not applicable were patients with an EMS-witnessed arrest and/or those that EMS decided not to transport

AED: automated external defibrillator, BLS: basic life support, BVM: bag-valve-mask, CPR: cardiopulmonary resuscitation, EMS: emergency medical services, ETT: endotracheal tube, IQR: interquartile range, SAD: supraglottic airway device, OHCA: out-of-hospital cardiac arrest, ROSC: return of spontaneous circulation, VF: ventricular fibrillation, VT: ventricular tachycardia.

Supplementary figure A – definitive airway device proportions, stratified by year of arrest
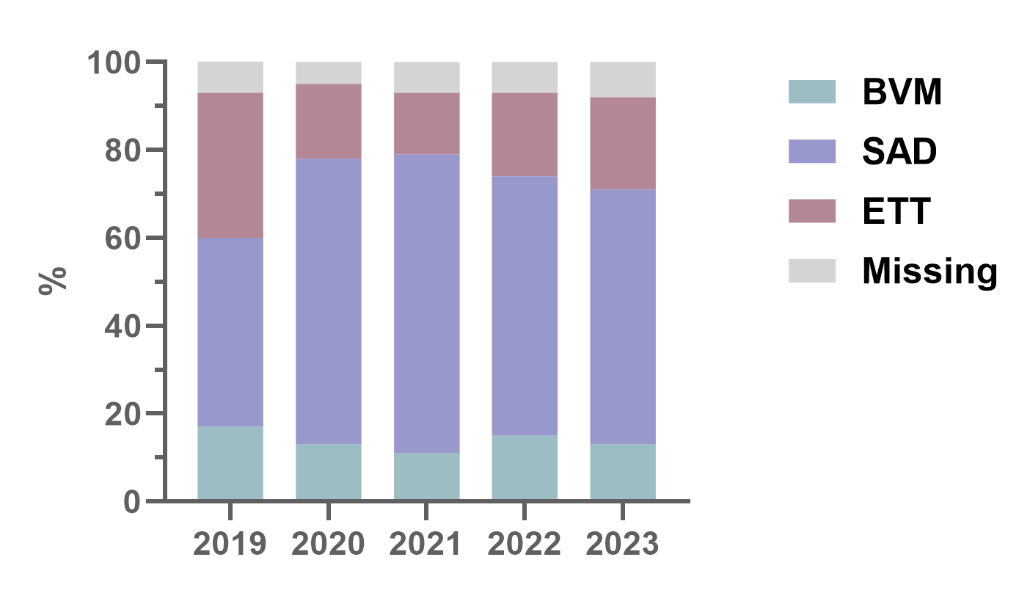


BVM: bag-valve-mask, ETT: endotracheal tube, SAD: supraglottic airway device
